# The Provincial Hospitals' Arrangements

**Published:** 1914-09-12

**Authors:** 


### The Provincial Hospitals' Arrangements.

(FROM OUR SPECIAL CORRESPONDENTS.)

### BRISTOL.

ON Wednesday, last week, some 130 wounded of the Royal Irish Rifles, Scots Fusiliers, Scots Greys, Royal Field Artillery, Gordon Highlanders, and other regiments who were among those landed at Southampton arrived at Bristol.

They were conveyed in a Great Western Railway special hospital train, and were received at Temple Meads Station by the Nos. 2, 4, 8, and 12 Women's Voluntary Aid Detachments and the Nos. 1 and 3 Men's Voluntary Aid Detachments and the St. John Ambulance Association, etc. Upon arrival refreshments were served to the soldiers, and the work of conveyance to the Royal Infirmary was quickly carried out by ambulances and private motor-cars. After examination the patients were allotted to the various wards. The cases are understood, on the whole, not to have been of a serious nature. Many of the wounded in passing through Bath Station threw addressed letters and cards out of the train, which were readily picked up by those standing on the platforms, and stamped and posted to the friends of the wounded soldiers.

### CAMBRIDGE.

THE few sick admitted into Addenbrooke's Hospital from among the troops now stationed in Cambridge and the neighbourhood have been removed to the Lees School, where a number of beds had been provided for use, and where other sick soldiers had also been admitted for treatment. On August 29 these sick soldiers were again moved, on this occasion from the Lees School to Trinity College, Cambridge, where Neville Court has been efficiently equipped as a hospital. The Cloisters have been converted into wards, and, although the arrangement is but temporary, a well-equipped operating-theatre is attached. On August 31 about 150 wounded soldiers arrived in Cambridge, and were conveyed to the 1st Eastern General Hospital at Trinity College, where they are now being attended by the principal matron, sisters, and nurses of the Territorial Force Nursing Service, and by the physicians and surgeons. On being conveyed from the station to the 1st Eastern General Hospital the wounded soldiers were given a most enthusiastic welcome by those who had assembled to witness their arrival.

For the most part the wounded are those who were in action at Mons, and in spite of their wounds they are very cheerful. For some days, with the consent of the authorities, many residents and others were allowed to visit the soldiers in hospital. The soldiers appreciated the exchange of conversation with the visitors, and the latter were more than interested in all that the soldiers were able to relate. As soon as it was known that a number of our soldiers who had been wounded in action had arrived at Cambridge gifts of all kinds flowed in, and among them Messrs. Gaymer & Son, Ltd., of Attleborough, Norfolk, sent for their benefit nine sieves of plums, two large barrels of apples, and three cases of cider. Some of the wounded have been subjected to $x$-rays in the $x$-ray department at Addenbrooke's Hospital. The patients generally are improving in health under the skilled treatment which they are receiving. The physicians and surgeons on duty at the 1st Eastern General Hospital are: Professor Sir Clifford Allbutt, K.C.B., M.D., F.R.C.S.; Professor J. B. Bradbury, M.D., F.R.C.P.; Laurence Humphry, M.D., F.R.C.P.; J. Aldren Wright, M.D., M.R.C.P.; G. Secretan Haynes, M.D., physicians; Joseph Griffiths, M.C., F.R.C.S., Colonel-in-Chief R.A.M.C.T.; F. Deighton, M.A., M.B., B.C.; George Wherry, M.C., F.R.C.S.; Major Apthorpe Webb, surgeons; W. Malden, M.D., M.R.C.P., pathologist.

A large number of young men from Cambridge have responded to Lord Kitchener's appeal, and have enlisted for service in the Second Army.

About 130 medical students were in the medical unit of the C.U.O.T.C. (Cambridge University Officers' Training Corps). They have received a comprehensive training organised by Major H. B. Roderick, M.D. Lectures were given to the students by Professor F. Howard Marsh, Professor Sims Woodhead, Dr. Barclay-Smith, Mr. Purvis, M.A., and Mr. Kempson, M.A. Having completed their field training these gentlemen have been sent to a number of London hospitals as temporary dressers in order to gain experience and enable them to qualify as dressers in clearing hospitals and hospital ships. About 300 students of the University have recently been examined by Major H. B. Roderick, M.D., for commissions in Lord Kitchener's Army.

There were last week many thousands of troops in Cambridge and district, and local people are providing comforts for them at the camps. At most of the camps there are recreation tents where the soldiers are able to read the daily papers and magazines, and also tents where cheap refreshments are obtainable. Many of the troops have now departed. General Keir has conveyed to the Mayor and to the residents of Cambridge his sincere thanks for the help and cordial assistance which they have accorded to him, to his staff, and to all under his command during their stay in Cambridge.

The National Relief Fund organised in the borough, etc., has reached in total over £4,000, and that in the county about £2,600.

The Borough Division of the British Red Cross Society have appealed for £250 to provide clothing for our soldiers, and already the appeal has produced about this sum.

Six hundred of the Cambridgeshire Territorials, under command of Colonel Tebbutt, have volunteered for foreign service, and a great effort is being made to bring the regiment up to war strength.

### CHATHAM DISTRICT: GERMAN PRISONERS.

St. Bartholomew's Hospital, Rochester, is now affiliated to the Central Military Hospital at Chatham, and the officer in charge of the Central Military Hospital has been directed to transfer wounded soldiers to St. Bartholomew's in direct consultation with the authorities. Six patients from Fort Pitt Hospital have been removed to Claremont House, the temporary hospital provided by the Strood and Frindsbury Voluntary Aid Detachment. About eighty wounded warrant officers and seamen were brought to Chatham Dockyard last week and conveyed in ambulances to the Royal Naval Hospital. There were amongst them about twenty wounded German naval prisoners, who were sent to Fort Pitt Hospital, Chatham. At the request of the General commanding the Thames and Medway defences, a small committee of owners of motor-cars has been formed to make arrangements with those owners who are willing to lend cars for the conveyance of wounded from ship to hospital, or from hospital to hospital, within the district of the command.

### GRAVESEND.

Every member of the staff of Gravesend Hospital has worked enthusiastically to help to get ready the extra beds which have been equipped for the sick and wounded, and all are now ready. The other side of the case—the dependants left behind—has also not been forgotten, for, acting on the suggestion contained in The Hospital, weekly collections have been instituted, and the dispenser, Miss Roberts, who acts as secretary and collects the contributions from the staff, sends each week through the Mayor the total to the Prince of Wales' Fund.

### IPSWICH.

Fourteen soldiers who were injured in the mobilisation are under treatment in the wards of the East Suffolk Hospital, Ipswich. At the time of writing no wounded sailors have been received.

### LEICESTER: ARRIVAL OF WOUNDED.

On September 2, within half-an-hour of the arrival of the Red Cross train at Leicester, 127 wounded and sick soldiers from the front were safely on the way to the 5th Northern General Hospital, so that it may be said that the local arrangements were a triumph of organisation. To reconstruct an old building which, built as a "lunatic asylum," had been disused for several years, and to make it habitable in the short space of two or three weeks in which the task had necessarily to be accomplished, was an immense undertaking, and it was with a wise foresight that Major Harrison, the commanding officer; Miss Vincent, principal matron; and Mr. S. P. Pick, the architect; and the hospital staff concentrated their efforts on having, at any rate, a portion of the building ready for occupation so as to cope with a sudden demand for beds. Consequently, when at about 5 a.m. on Wednesday, last week, word was received that wounded soldiers to the number of about 130 would be entrained at Southampton that day, everything was in readiness and there was no hitch of any kind. About four o'clock the train steamed into the station, the wounded men being under the care of Captain Stracey, of the North Midland Clearing Hospital. Here were assembled Mr. A. W. Faire, the County Director of the Voluntary Aid Detachments, and Mr. W. F. Seymour, his assistant; Lieut.-Colonel Pemberton Peake was in charge of the platform party, and Major L. K. Harrison. A fleet of motor-cars had been arranged by Mr. P. L. Baker, Mr. J. R. Corah, and others, and within half-an-hour the last of the wounded men had left the station. At the 5th Northern General Hospital all was in readiness for the men, and tea was provided on arrival. The resident matron, Miss Hamath, has now a staff of thirty-one trained nurses under her, and arrangements have been made for the immediate provision of a further staff in the not unlikely event of more patients being received. Generally speaking, the wounds of the men were not of a serious nature. Quite a big proportion were cases for medical treatment, cases of ulcerated feet and so forth. The final touches are now being put to the building, and it is anticipated that the full complement of 520 beds with the requisite officers will be available in a few days. The occupation of the hospital by wounded troops has been the means of encouraging gifts of all kinds, including a plentiful supply of newspapers given by the local press. At the time of writing the Mayor of Leicester's Fund for sick and wounded soldiers and their dependants amounts to upwards of £17,000.

### LEAMINGTON SPA.

Two of the honorary officers of the Warneford Hospital, Leamington, Dr. Sydenham and Dr. Clayton, have been mobilised with the Territorial Forces, and Dr. H. E. Creswell, house physician, has joined the R.A.M.C. Dr. A. L. Davies retains his post as house surgeon, and Dr. Gibbons Ward, the medical officer of health, has volunteered his services as visiting house physician. Two members of the nursing staff have joined Q.A.I.M.N.S., one being stationed on Salisbury Plain and the other having been sent with the Expeditionary Force to France. One nurse has also joined the Territorial Forces at Southern Command Hospital, Birmingham. The local Victoria Park is closed to the public, as it is being used as a veterinary hospital in connection with the Southern Command.

### MANCHESTER MEN ON ACTIVE SERVICE.

At the Manchester Children's Hospital, Pendlebury, the senior surgeon, Mr. Charles Roberts, M.B., F.R.C.S. Eng., has been called up for active service with the T.F., R.A.M.C. (Bearer Company). Dr. C. Paget Lapage, M.D., M.R.C.P., has been called up for duty at the headquarters of the Manchester University Officers' Training Corps. Miss E. A. Renaut, the matron, has joined her unit of the Territorial Nursing Service at St. Gabriel's College, Camberwell.

Dr. J. H. Wright, second assistant medical officer at the out-patients' department, who is a member of the R.N.R., has left to take up duties as a surgeon in the Navy, and Dr. Thomas Hayhurst, the senior resident medical officer at the hospital, has left to take up duty as a surgeon in the Army. The sister of the Victoria Ward and the staff nurse are waiting their orders to join the Naval Nursing Service and T.F. respectively.

### MIDDLESBROUGH.

Immediately on the mobilisation of H.M. Forces the North Riding Infirmary, Middlesbrough, made arrangements to place sixty beds at the disposal of the military authorities, in conformity with an agreement made some years ago, and all the local arrangements for the transport of sick or wounded to this hospital have been completed. The normal work of the infirmary is proceeding as usual, with the exception that only cases of accident and emergency are being admitted to the male wards.

All cases needing medical attention, which occur amongst the troops stationed at the mouth of the Tees, are being sent to the infirmary for treatment, and already a number of cases have been dealt with. In this connection, the infirmary's horse-ambulance service has proved of great use in the transport of military patients to the institution. The colonel in command of this district paid a visit of inspection to the infirmary a few days ago, and expressed himself as being highly satisfied with the arrangements.

#### North Ormesby Hospital.

The Red Cross detachment in the Cleveland district are very anxious that their arrangements for the reception of the wounded shall be as complete as possible, and with this object several of their nurses are attending the North Ormesby Hospital as paying probationers, in order to obtain practical experience. The Royal Naval Friendly Union of Sailors' Wives has asked Mr. E. C. Smith, the secretary of North Ormesby Hospital, to act as hon. secretary of the Royal Naval Medical Information Bureau for the Middlesbrough and Cleveland district. Mr. Smith had agreed to do this, but with his appointment as secretary of the Queen's Hospital, Birmingham, he will be compelled to withdraw from the work.

### NORWICH.

The offer of the Board of Management of the Norfolk and Norwich Hospital to place 250 beds at the disposal of the Admiralty has been accepted, as has also their offer of 100 beds to the War Office. The institution, at the time of writing, has not received any wounded from the seat of war, but one of its wards has been occupied by soldiers who have been disabled through marching and other causes.

### THE TEMPORARY WARDS AT NEWCASTLE.

In view of the need for further accommodation in connection with the Royal Victoria Infirmary, Newcastle, two temporary wards have been put up. Various offers of accommodation from private houses which were scattered all over the place had been received, but the hospital authorities thought it much better to save both money and energy by concentration, and they have had no difficulty in raising the sum required. They have, of course, tried not to spend a farthing unnecessarily. The people in the district have been most generous in offering accommodation for hospital purposes, but a great many of the offers are really unsuitable, entailing too great difficulties connected with nursing, etc., to justify their acceptance. Lord Ridley most kindly placed thirty beds at Blagdon at the hospital's disposal for civil patients, and several were sent there, but this accommodation has now been transferred to the military authorities for convalescent wounded. No wounded have at the time of writing yet been sent to the Royal Victoria Infirmary. The accommodation at the disposal of the authorities has been utilised for Territorials, of whom there are at present between 300 and 400 in hospital.

The following is a description of the two temporary wards, both of which are the same length—namely, 102 feet 6 inches. They are simply an elongation of the existing wards, and access to them must be through the existing wards. The new wards will also have to use the same sanitary towers and bathrooms, this limitation being, of course, for the sake of cheapness. The wards stand on timber foundations; the walls are of weather boarding, lined with match-boarding, with a layer of felt between; the roof is of corrugated iron. The end farthest from the entrance will have a large window and a large door, which can be opened in case of necessity. There will be a window between each bed. The space at the entrance to the ward will be fitted up as convenience dictates; one of the spaces will have to be a service-room. The authorities have been obliged to put up the simplest and cheapest construction possible. The wards are not placed exactly in a line with the existing blocks, but are staggered to one side. This is to obstruct as little as possible the large open doors at the end of the existing wards. They will be heated by two calorifiers placed in a sunken chamber at the side of the ward, heated by steam from the hospital's boilers. While they do not pretend to be more than uninteresting temporary structures, they will be, at any rate, well ventilated and warm. They are, of course, of a purely temporary character.

With these two extra wards and with the offer of forty-two beds at the Home for Incurables, where the accommodation from a hospital point of view is excellent in every respect, the Royal Victoria Infirmary will be able to maintain for the civil population 400 beds, and hand over to the military authorities 200 for their cases.

The hospital authorities are working in the closest conjunction with the 1st Northern General Hospital in Armstrong College, which is now fairly established. This 1st Northern General Hospital will have at its disposal in a short time 1,000 beds. At least, this number is semi-officially stated. As to the new wards, the Royal Victoria Infirmary authorities know there are many points to criticise. Access to the existing wards could not be avoided. It was much desired not to have anything between the existing blocks, as any structure so placed would interfere with the free passage of air. By placing them at the end the hospital committee feel they have done no harm to the circulation of air, but there is, of course, an awkward access.

### SHEFFIELD: WOUNDED FROM MONS.

At the Royal Hospital two wards in the Princess of Wales block—the Keeling and Edgar Allen wards—have been placed at the disposal of Col. Connell. They will provide from thirty-six to forty beds. The annexe at Fulwood, with forty-two beds, has also been placed in the hands of the military authorities.

The hospital arrangements made in Sheffield for dealing with war casualties were called upon for the first time last week. On Wednesday, September 2, 125 soldiers, belonging to a great variety of regiments, who had been wounded in the four days' fighting around Mons, arrived in the city from Southampton, and were taken to the 3rd Northern General Hospital in Collegiate Crescent. They were met at the station by Lieut.-Colonel Connell and Captain Pooley, with a staff of men, and all were transferred from the hospital train to the motor 'buses and ambulances in waiting in half-an-hour. There were few serious cases, and nearly all the men were able to walk to the Corporation motor 'buses, lent by the Tramways Committee, in which they rode to the hospital. The Sheffield Education Committee's Training College and Women's Hostel, which had been ready for the reception of wounded for some weeks past, form a most useful hospital. The large, lofty, and well-lighted rooms make good wards, and there is a large and efficient staff of doctors and nurses, the latter being under the direction of Miss Earle, matron at the Royal Hospital. The wounded men are making good progress. The City Council have decided to place five wards at the Lodge Moor Fever Hospital at the disposal of the military authorities. It is proposed to erect tents for convalescent scarlet fever and diphtheria cases. These arrangements will provide for the reception and treatment of 150 military cases without in any way curtailing the accommodation for civilian cases, and they are considered preferable to accepting the generous offers of private individuals to place buildings at the disposal of the committee, as a more thorough control and observation of cases can be exercised. In order to make these arrangements, none except the very urgent cases were removed into hospital for a period of six days, between August 6 and 13. The Mappin Art Gallery, a public collection of pictures belonging to the Corporation, has also been scheduled for hospital purposes. The gallery is situated in the fresh air and among the pleasant surroundings of one of the public parks, and the rooms are large and well ventilated. If it is used as a hospital it will be necessary to remove most of the valuable collection of pictures.

### TUNBRIDGE WELLS AND DISTRICT.

The hospital at Tunbridge Wells recently received a letter from the War Office asking upon what terms troops would be received into the General Hospital. The reply was to the effect that treatment, medical and surgical, nursing, etc., would be quite free, but that the committee would be glad to receive a grant to defray the cost of rations. The War Office replied accepting the hospital as a section of the Central Military Hospital at Chatham, so troops may be expected at any time. The General Hospital was inspected by a medical officer from Chatham on Thursday, September 3, and the accommodation for the fifty sick or wounded troops which the committee had undertaken to care for was approved of. In a short time cases will be sent, and arrangements have been made with the local detachment of the St. John Ambulance Society to transport the patients from the railway station to the hospital. Two more sisters have been summoned to Brighton from the General Hospital, Tunbridge Wells, for duty in the Territorial Nursing Service.

